# 3D strain-induced superconductivity in La_2_CuO_4+δ_ using a simple vertically aligned nanocomposite approach

**DOI:** 10.1126/sciadv.aav5532

**Published:** 2019-04-26

**Authors:** Eun-Mi Choi, Angelo Di Bernardo, Bonan Zhu, Ping Lu, Hen Alpern, Kelvin H. L. Zhang, Tamar Shapira, John Feighan, Xing Sun, Jason Robinson, Yossi Paltiel, Oded Millo, Haiyan Wang, Quanxi Jia, Judith L. MacManus-Driscoll

**Affiliations:** 1 Department of Materials Science & Metallurgy, University of Cambridge, Cambridge, UK.; 2 Sandia National Laboratories, Albuquerque, NM 87185, USA.; 3 Racah Institute of Physics and Center for Nanoscience and Nanotechnology, The Hebrew University of Jerusalem, Jerusalem 91904, Israel.; 4 Department of Materials Engineering, Purdue University, West Lafayette, IN 47907, USA.; 5 Department of Applied Physics and Center for Nanoscience and Nanotechnology, The Hebrew University of Jerusalem, Jerusalem 91904, Israel.; 6 Department of Materials Design and Innovation, University at Buffalo—The State University of New York, Buffalo, NY, USA.

## Abstract

A long-term goal for superconductors is to increase the superconducting transition temperature, *T*_C_. In cuprates, *T*_C_ depends strongly on the out-of-plane Cu-apical oxygen distance and the in-plane Cu-O distance, but there has been little attention paid to tuning them independently. Here, in simply grown, self-assembled, vertically aligned nanocomposite thin films of La_2_CuO_4+δ_ + LaCuO_3_, by strongly increasing out-of-plane distances without reducing in-plane distances (three-dimensional strain engineering), we achieve superconductivity up to 50 K in the vertical interface regions, spaced ~50 nm apart. No additional process to supply excess oxygen, e.g., by ozone or high-pressure oxygen annealing, was required, as is normally the case for plain La_2_CuO_4+δ_ films. Our proof-of-concept work represents an entirely new approach to increasing *T*_C_ in cuprates or other superconductors.

## Teaser

We demonstrate a new way to increase the superconducting *T_C_* of cuprates using 3-D strain control in nanocomposite thin films.

## INTRODUCTION

High-temperature superconductor (HTS) cuprates are very important materials for a range of energy applications, and increasing their superconducting transition temperature (*T*_C_) is an important goal. There are a range of cuprates with *T*_C_ values above 100 K, even up to >150 K in Hg-based compounds under pressure (*1*), but their structures and processing are highly complex, and most cuprates have strong electric anisotropy, which means that their in-field properties are poor. Increasing the *T*_C_ value of single-layer cuprates could lead to strong benefits for practical applications, in terms of both performance and cost (*2*).

From a phenomenological perspective, the methods for achieving high *T*_C_ in the cuprates are well known (*3*): (i) optimize the carrier concentration in the CuO_2_ planes (*4*); (ii) eliminate defects and disorders in the CuO_2_ planes (*5*, *6*); (iii) make the planes flat, square, and of optimal size [superexchange coupling of Cu–O–Cu in the planes correlates with the superconducting order parameter; the *a* parameter (*a*) should be optimized] (*7*, *8*); (iv) optimize the interplanar distance; and (v) have a large Cu-apical oxygen (Cu-O_A_) distance to ensure that carriers are localized in the CuO_2_ planes [the *c* parameter (*c*) should be maximized] (*9*–*11*).

**Fig. 1:**
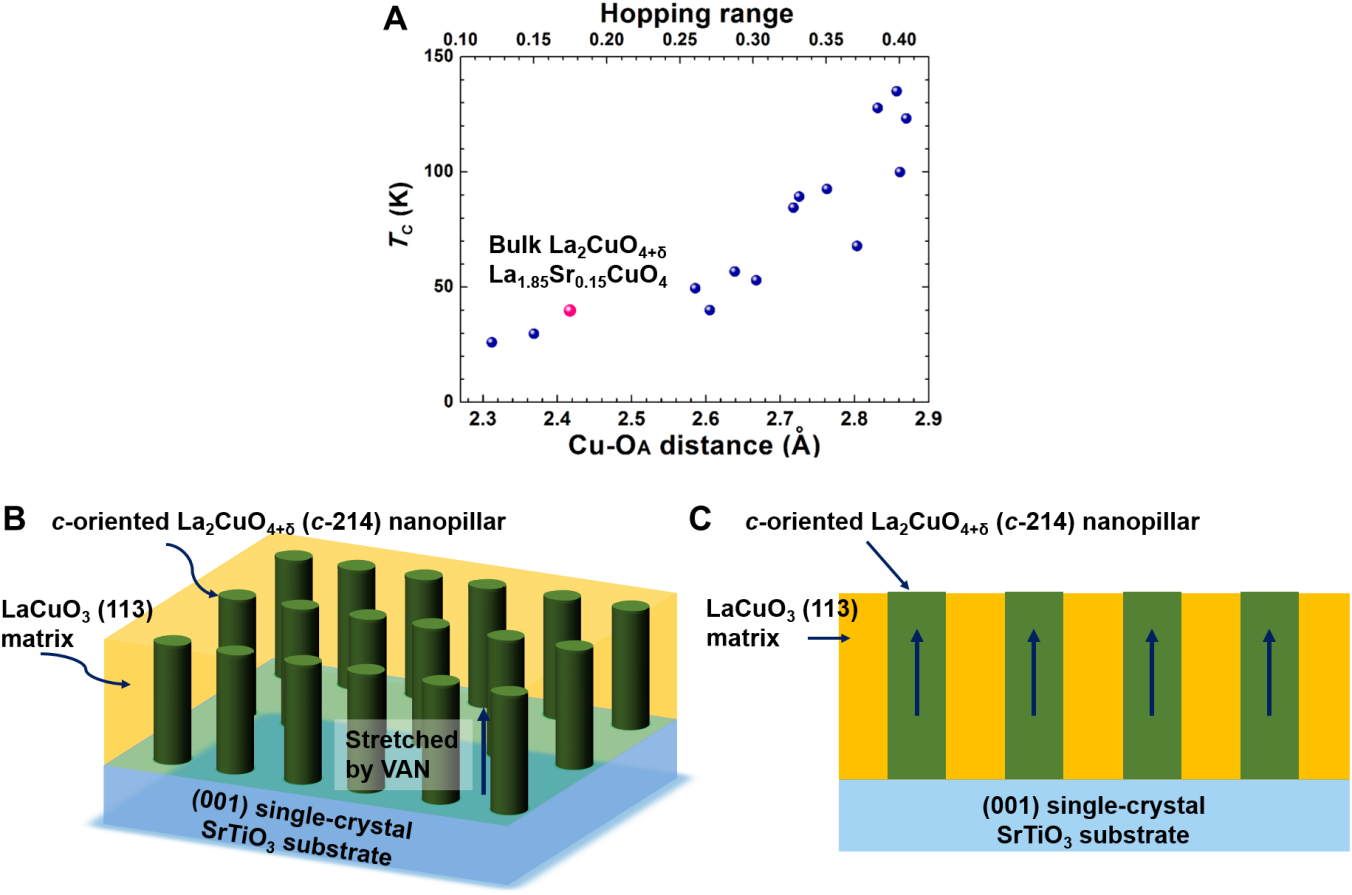
*T*_C_ versus Cu-O_A_ distance/hopping range istance for cuprate superconductors and the standard VAN structure. (**A**) *T*_C_ versus Cu-O_A_ distance/hopping range for one-, two-, and three-layer cuprate systems [main data from (*20*)]. La_2_CuO_4+δ_ and La_2–*x*_Sr*_x_*CuO_4_ both have maximum *T*_C_ values of 40 K in bulk, as indicated on the plot by the red point. The calculated correlation between *T*_C_ and the hopping range (the range where holes can hop in the CuO planes), which is linked to the Cu-O_A_ distance, is shown on the top axis (*20*). A larger hopping range and a weaker contribution of the d_z2_ orbital of Cu give more localized conduction (more 2D-like) in the CuO_2_ layers (*9*–*11*, *20*), and these lead to the higher *T*_C_. The standard VAN structure aimed for in this work is composed of a LaCuO_3_ film matrix, with La_2_CuO_4+δ_ nanopillars incorporated by self-assembled growth, as shown in three-dimensional (3D) (**B**) and 2D views (**C**).

For point (v), the close relationship between the *T*_C_ and Cu-O_A_ distance has been demonstrated experimentally by chemical substitution of cations (*6*, *12*–*14*), application of hydrostatic pressure (*15*–*17*), and control of Madelung (electrostatic) potential (*18*, *19*). Figure 1A shows the strong dependence of *T*_C_ on the Cu-O_A_ distance and the charge carrier hopping range for hole-doped cuprates, including one-, two-, and three-layer systems (*11*, *20*).

In the (La,Sr)_2_CuO_4+δ_ system, in-depth studies of interfaces have been instrumental to control Madelung strain, electronic reconstruction, and mobile charge carrier concentration (*18*, *19*, *21*–*24*). (La,Sr)_2_CuO_4+δ_ is an ideal system to study the tuning of the lattice in relation to *T*_C_, because it has a relatively low variation of excess oxygen content (i.e., 0.05 < δ < 0.13) and is less likely to lose oxygen in response to strain compared to, for example, YBa_2_Cu_3_O_7–*x*_.

The landmark work of metal/superconductor or metal/insulator bilayer La_1.55_Sr_0.45_CuO_4_/La_2_CuO_4+δ_ films grown on LSAO (LaSrAlO_4_) showed a strong link between *T*_C_ and Cu-O_A_ in La_2_CuO_4+δ_. *a* was controlled by epitaxial strain with the substrate, and *c* (and hence Cu-O_A_) was controlled by Madelung electrostatic strain (out-of-plane Coulomb interactions) (*18*, *19*, *25*). *T*_C_ was increased to 50 K at the bilayer interface in a single unit cell (*18*, *19*). Doping studies, in addition to Madelung strain, also showed the importance of lattice expansion on *T*_C_ (*14*). The bilayer thin film work confirmed previous uniaxial mechanical tensing (8 GPa) along the *c* direction in La_1.85_Sr_0.15_CuO_4_ single crystals, where *T*_C_ was increased to 52 K from ~40 K (*17*). We note that strain effects have also been shown to be important in FeSe superconductor films and, with *T*_C_, increased by up to 65 K from 8 K at the film/substrate interface (*26*).

It is important to note, however, that while LSAO is a very standard substrate for growth of the (La,Sr)_2_CuO_4+δ_ films, whether in heterostructures or plain films, it is not an ideal substrate from a structural matching viewpoint (*19*) because *a* = 3.755 Å, which is much smaller than the optimum value (~3.84 Å) to achieve a maximum *T*_C_ (*7*, *8*, *27*). Hence, point (iii) in the above list of methods for HTS is not optimized for epitaxial growth of single-layer cuprates on LSAO. The phenomenological relationship between the maximum achievable *T*_C_ obtained for a wide range of cuprate superconductors and *a* is shown in Fig. 4E.

It is also important to note that the standard epitaxial thin films of La_2_CuO_4+δ_ have a lower *T*_C_ value than the bulk value (~40 K). Despite the nonoptimized lattice structure of LSAO, the highest *T*_C_ La_2_CuO_4+δ_ films are grown on this substrate; hence, a *T*_C_ value of ~35 K (5 K lower than the bulk value) is achieved (*19*). Only Naito *et al*. (*28*) presented a much enhanced *T*_C_ of 57 K in films of La_2_CuO_4+δ_ grown on LSAO. They postulated that this high *T*_C_ was due to the epitaxial strain. However, this result was never reproduced, although the epitaxial strain effect on superconductivity has been widely explored (*19*, *28*–*30*). Lower *T*_C_ values are obtained on other substrates (*31*), and this is likely related to disorder effects in addition to non-optimized strain effects. The use of epitaxial strain to tune the strain in plain La_2_CuO_4+δ_ films is not ideal because it is not possible to simultaneously optimize *a* and *c*, i.e., they cannot be controlled independently owing to Poisson/elastic effects (*19*, *32*). Hence, points (iii) and (v) in the above list of the methods for HTS will not be optimized simultaneously.

The question to be explored in this work is whether it is possible to increase *c* in the (La,Sr)_2_CuO_4+δ_ system without reducing *a*, which is the case when the standard LSAO substrate is used, and therefore whether it is possible to further increase *T*_C_. The work also explores whether it is possible to achieve more than just a single unit cell effect. As described below, the approach used is to engineer the lattice strain in a new way so that the Cu-O_A_ distance is increased and the in-plane distance is not reduced compared to bulk.

We use a vertically aligned self-assembled (i.e., naturally formed) nanocomposite (VAN) system in which the interfaces are clean, i.e., there are no second phases or chemical mixing, which can occur with artificial superlattices (*33*). The standard common VAN structure is shown in Fig. 1 (B and C). Our aim is to create a VAN structure consisting of vertical La_2_CuO_4+δ_ nanostructures, e.g., standard nanopillars, in a LaCuO_3_ matrix. We chose to use pure La_2_CuO_4+δ_, and not La_1.85_Sr_0.15_CuO_4_ [(Sr)214], because Sr would also substitute in LaCuO_3_, which would lead to suboptimal and, more importantly, unspecified doping in La_2_CuO_4+δ_. We chose LaCuO_3_ as a strain-controlling phase because it will not poison La_2_CuO_4+δ_ and contains the same chemical constituents. In addition, because LaCuO_3_ contains Cu^3+^, it can act as an oxidizing source to dope La_2_CuO_4+δ_ (*34*). Under the film growth conditions, LaCuO_3_ will most likely be in the composition range, LaCuO_3–δ_, 0 < δ < 0.5, which will make it antiferromagnetic (*35*, *36*). In our pure La_2_CuO_4+δ_ system, optimal hole doping then relies on obtaining highly oxygenated La_2_CuO_4+δ_.

In a VAN structure, the La_2_CuO_4+δ_ nanostructured inclusions will be stretched along the out-of-plane *c* direction by vertical epitaxy with the LaCuO_3_ matrix. We do not use chemical substitution for inducing strain as this simultaneously expands (or contracts) both the out-of-plane and in-plane lattice parameters. In addition, we do not use substrate control of *a* to induce an elastic expansion of *c* (and hence the Cu-O_A_ distance) because this will require shrinkage of *a*, which is undesirable. We use a (001)-oriented SrTiO_3_ (STO) substrate to enable La_2_CuO_4+δ_ to keep its bulk *a*. This is the case because La_2_CuO_4+δ_ does not grow coherently on STO owing to the different lattice structures (*31*).

The VAN approach is very different to the heterostructure Madelung strain approach because (i) the in-plane parameter should be maintained at the bulk value and is not reduced by growth on LSAO; (ii) it is relatively simple as films are grown from a composite target; (iii) it allows growth of billions of interfaces per film rather than just one or a few, giving the possibility to increase *T*_C_ of the whole film if the interfaces are closely spaced enough; and (iv) the vertical strain in the La_2_CuO_4+δ_ nanopillars is highly tunable by choosing different matrix materials.

Here, La_2_CuO_4+δ_ is referred to as 214 and LaCuO_3_ is referred to as 113. Although detailed elastic moduli are not available for both 113 and 214, 113 is assumed to be stiffer than 214 along the *c* direction because the layered 214 structure should be more compliant along the *c* direction. Hence, the hypothesis is that 113 will dominate the strain state in 214 via vertical epitaxy.

We note that while 113 has the same basic building blocks as 214, i.e., the LaCuO_3_ perovskite units contain CuO_6_ octahedra, it is nonsuperconducting, likely because it contains a three-dimensional (3D) network of O–Cu–O bonds (*35*). Depending on its oxygen content, 113 ranges from a poor metal to an insulator to a semiconductor (*36*). In terms of its magnetic properties, 113 has a rich magnetic phase diagram showing antiferromagnetism (AFM), Pauli paramagnetism, or AFM with ferromagnetic canting, depending on the oxygen content (*36*).

More than 10 epitaxial, self-assembled thin films of 214 and 113 (thickness, ~25 to 100 nm) were grown from a single mixed ceramic target by pulsed laser deposition (PLD) onto (001) STO substrates. STO was chosen as the substrate as it has a perovskite structure similar to that of 113, and so, 113 will be epitaxially stabilized on STO. 113 has a tetragonal structure (*a* = 3.8189 Å; *c* = 3.97268 Å; unit cell volume, 57.993 Å^3^) when it is fully oxidized (*34*–*36*). Because *c* is most closely matched to *a* of STO (*a* = 3.905 Å), the films are expected to grow with the out-of-plane *a* axis, giving *a* axis–oriented 113 films (*a*-113). As already mentioned, because 214 is structurally mismatched to STO, this means that 214 will not grow coherently on the STO and so should be relaxed in plane (*31*, *32*). From the point of enhancing *T*_C_, a relaxed *a* for 214 (i.e., 3.79 Å) is preferable over a reduced one, as is the case when it is grown epitaxially on LSAO (i.e., 3.755 Å).

We used a composite PLD target of 214:113 with an atomic ratio (*n*:*m*) of 2:3. There is a good stoichiometry transfer from target to films using PLD, and so, the atomic ratio was assumed to be the same in the films and target. We aimed to achieve relatively fine nanostructures of *c* axis–oriented 214 (*c*-214) inclusions in the 113 matrix so that, as much as possible, 214 would be strained vertically by 113 without relaxing laterally to the bulk value. A smaller fraction of 214, and hence a lower *n*/*m* value, would give finer nanostructures (*33*), but a very small value means that the nanostructures would become discontinuous.

All films, except for one, labeled S5, were grown using the same growth and annealing conditions as described in Materials and Methods. S5 was cooled without post-annealing in O_2_ after growth.

We show that *T*_C_ can be increased to ~50 K in 214 VAN films, consistent with the observation of >10 nm highly strained (expanded *c* without reduction) regions around the 214/113 interfaces, in which there are billions in each film, i.e., in much larger volumes than in previous artificial superlattice studies. More than 10 films were grown with enhanced *T*_C_. We note that 214 films or La_1.85_Sr_0.15_CuO_4_ [(Sr)214] films of similar thickness grown on STO are not superconducting [this work, (*32*)] and highly optimized films grown on LSAO have *T*_C_ values of ~35 K (*19*). Our work shows that the increased *T*_C_ is closely linked to increases in *c*, while *a* lies close to the bulk value. It also shows that the VAN approach eliminates the need for post-annealing under highly oxidized conditions. We propose how to increase the volume fraction of the 50 K phase in the VAN films and how to further increase *T*_C_.

## RESULTS AND DISCUSSION

We focus on the properties of the five ~25-nm-thick films (films S1 to S5). Films of 50 nm thick and above did not provide reliably increased *T*_C_ values. As we will show later, this is related to strain relaxation of the strain-controlling *a*-113 phase.

We show that simple O_2_ annealing, rather than O_3_ annealing, as is normally the case for plain 214 films, is sufficient to provide hole doping for superconductivity. We further show this using x-ray photoemission spectroscopy (XPS) on two nanocomposite films: one cooled without post-annealing in O_2_ after growth (S5) and the other with post-annealing at a *P*O_2_ (partial pressure of oxygen) of 500 mbar for 1 hour after growth (S3).

As shown in note S1, the valence band (VB) spectra for both samples consist of strongly hybridized states of Cu3d and O2p. S3 shows a metallic state as confirmed by the appearance of a density of states across the Fermi level, whereas S5 is insulating. The VB spectrum of S5 is in agreement with the insulating phase of 214. The metallic state of S3 is consistent with a hole-doped induced insulator-to-metal transition, i.e., excess oxygen by annealing in oxygen.

Further evidence of excess oxygen is confirmed by the concurring shift of the binding energies (BEs) of La 4d and O1s toward lower values of S5. This BE shift is due to a downward shift of the Fermi energy (chemical potential) by hole doping, which has been observed in many hole-doped transition metal oxides and hole-doped cuprate oxides (*37*). Hence, S3 was oxygenated without carrying out super-oxygenation or ozone annealing after growth, as is normally the case to make 214 superconducting (*19*, *30*, *32*). In addition, with 113 as an oxygen source in the film, local stretching of *c* in 214 may also enable easier oxygen incorporation into interstitial positions, as we will discuss later.

**Fig. 2:**
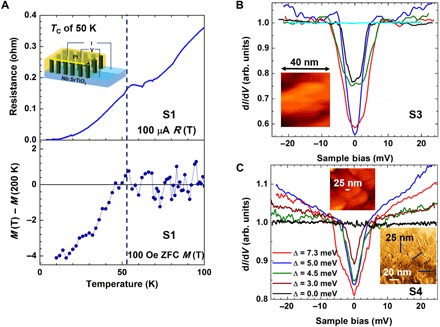
Measurements of superconductivity in films S1, S3, and S4. (**A**) Resistance versus temperature, *R* (T), measured in a four-probe configuration during warming with bias current *I* = 100 μA (top) and corresponding ZFC temperature dependence of the dimensionless magnetic moment (bottom), *M* (T) – *M* (200 K), for the same thin film at *H* = 100 Oe. The inset of the top panel shows the top-to-bottom measurement geometry. (**B**) Differential conductance d*I*/d*V* versus applied voltage *V* tunneling spectra measured (except the cyan curve) at 4.2 K, demonstrating the large variation of gap widths (Δ) and depths. The inset shows a topographic image of an area where the blue (Δ = 5 meV) and red (Δ = 10 meV) spectra were acquired. The cyan curve was measured at 78 K, well above *T*_C_, showing no superconductivity-related features. (**C**) Spatial evolution of proximity-induced superconducting gaps in an Au-coated film measured across the 25-nm-long white line drawn on the STM topographic image, as shown in the top inset. The lower inset image (planar TEM image of the film surface) shows possible scan regions, which cross dense regions of the film, i.e., there are no pores in the film.

**Fig. 3:**
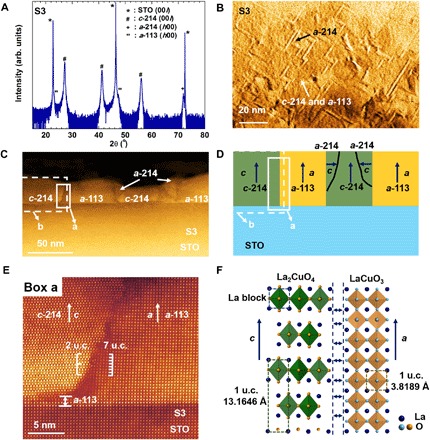
Microstructure, alignment, and structural analyses of 214:113. (**A**) XRD 2θ-ω spectra showing coexistence of *c*-214 and *a*-113. In addition, a small amount of *a*-214 is detected. (**B**) Plan-view TEM image showing an orthogonal pattern of *a-*214 grains embedded in the main film matrix. (**C**) Low-magnification cross-sectional STEM image. (**D**) Schematic structure of the film in (C). The white-boxed regions, a and b, represent interfacial regions that are studied further by high-resolution STEM and lattice parameter analyses. (**E**) High-resolution STEM image of the *c*-214/*a*-113 interface [white-boxed region a of (C) and (D)]. Crystal matching of two unit cells (u.c.) of *c*-214 with seven unit cells of *a*-113 is observed. (**F**) Schematic structural model showing two unit cells of *c*-214 matching with seven unit cells of *a*-113. The unit cells of 214 and 113 are outlined. The out-of-plane lattice parameters quoted are the bulk values. The La pseudo-cubic block in 214 is also outlined. The CuO_6_ octahedra, but not the individual Cu atoms, are shown.

We now focus on the superconducting properties of the films. Figure 2A shows the temperature dependence of resistance, *R* (T), for film S1, with a bias current *I* = 100 μA. A *T*_C_ onset of ~50 K is observed with a broad transition. The measuring geometry is from top to bottom, i.e., from the top electrode [platinum (Pt)] to the bottom electrode (Nb:STO substrate), as shown in the inset of Fig. 2A. It was necessary to do a top-to-bottom measurement because a lateral superconducting path would be blocked by the 113 phase. Room temperature conductance atomic force microscopy demonstrates the film structure of conductive regions embedded in insulating ones and is presented in note S2. The dependence of *R* (T) on the bias current (*I*) for film S2 is shown in note S3. One or more factors could be responsible for the broad superconductor transition: (i) The superconducting path may not be continuous from the top to the bottom of the film, leading to percolation effects that can broaden the transition. As shown in Fig. 3E, the *c*-214/*a*-113 interface is stepped, and so, the superconducting path could be tortuous. (ii) A very thin (two to three unit cells) insulating tunneling layer of *a*-113 is present at the interface between the *c*-214 film and the STO (also observed in Fig. 3E and detailed in note S3). (iii) The superconducting regions are filamentary and characterized by different *T*_C_ onsets due to an inhomogeneous strain distribution at the *c*-214/*a*-113 sample interfaces (*38*–*40*).

The electrically measured *T*_C_ onset of 50 K agrees with the temperature where the diamagnetic Meissner component becomes dominant in the zero-field cooled (ZFC) *M* (T) curve, as shown in the bottom of Fig. 2A. The Meissner signals were approximately few × 10^−6^ electromagnetic units at 10 K, indicating a ~5% superconductor volume fraction in the film.

To further explore the spatial variation of superconductivity, we undertook low-temperature (4.2 K) scanning tunneling microscopy (STM) and spectroscopy (STS) measurements on films S3 and S4 (that had a gold capping layer). The highly resistive 113 regions (at 4.2 K) prevented us from performing stable large-scale topographic images on sample S3 and achieving clear topography-spectroscopy correlations due to the many tip-sample crashes we encountered. To overcome this problem, we prepared a gold-coated (S4) sample, where correlations between topography and proximity-induced superconductivity were achieved. Let us first address the STS measurements performed on film S3. Superconducting gaps (Δ) in the differential conductance (d*I*/d*V*) versus applied voltage (*V*) tunneling spectra were found in localized areas. The gap values (Δ) and depths (zero-bias conductance) largely varied over the sample, the first from ~10 to ~2 meV and the second from ~0.55 to ~0.95 of the normal tunneling conductance, as demonstrated in Fig. 2B.

The quasi-particle coherence peaks observed in our tunneling spectra are smeared and much smaller compared to those typically observed in spectra measured on conventional superconductors, as well as on some of the high-temperature cuprate superconductors such as Bi_2_Sr_2_CaCu_2_O_8_ (Bi2212). Our results, however, are consistent with measurements performed on optimally doped (Sr)214 single crystals (*T*_C_ of 40 K) after cleaving under He gas (*41*). According to Kato *et al*. (*41*), in an optimally doped (Sr)214 single crystal, over 70% of the spectra have no apparent coherence peaks, and the magnitude of the observed peaks (if they exist) is much weaker than that typically observed for Bi2212. In our experiment, the coherence peaks may be further reduced because of spatial averaging in our nonhomogeneous sample and/or degraded surface conditions resulting from sample transfer from the growth chamber to the STM apparatus. In that respect, we note that STM/STS is a surface-sensitive tool (*42*), even to the level of identifying surface order parameter symmetry differing from that in the bulk (*43*), and therefore, signatures of d-wave superconductivity (which may exist in the bulk of our cuprate samples) can be reduced in the STM tunneling spectra due to surface disorder (*44*). However, d-wave superconductivity is still observed, although in a subtle way, by the in-gap spectral structure observed in the green and black curves in Fig. 2B (resulting in relatively high zero-bias conductance), manifesting the effect of zero-energy Andreev-bound states.

Moreover, we could fit well our STS data to spectra calculated using the theory for tunneling into a d-wave superconductor (*45*) over a wide range of tunnel barrier strengths, as detailed in notes S4 and S5. We also discuss in note S4 the correlation between the topographic image presented in the inset of Fig. 2B and the red and blue spectra. A maximal observed gap value of ~10 meV is close to that of optimally doped (Sr)214 single crystals (*T*_C_ of 40 K) measured after cleaving under He gas (*41*). Because our films were not vacuum-cleaved, their surfaces may have degraded, leading to a reduction in *T*_C_. Hence, the 50 K measured from the *R* (T) data is broadly in agreement with the maximal 40 K related measured gap by STS. As shown in Fig. 2B, much narrower gaps were also observed in different regions of film S3, indicating regions of lower *T*_C_ (<40 K) material (*41*) or highly degraded surface.

Further evidence of a spatial gradient of superconductivity was gained by studying the Au-capped film S4. The Au capping layer enables good measurement stability to be achieved over large scan areas, which contain regions of very low conductivity within which superconducting islands are embedded, as shown in (*46*). In our sample, proximity-induced superconducting islands develop in the Au film in regions that are directly connected to superconducting regions in the underlying film, and their spatial extension can be monitored by STS. Figure 2C demonstrates such measurements. The red curve was measured at the middle of the line, the green and blue curves were measured on the left, and the brown and black curves were measured on the right. It is important to note that we do not know the underlying surface morphology, so we can only speculate that the red curve was measured near the 113-214 boundary (showing the widest and deepest gap), the green and blue curves on the 214 nanostructure region, and the brown and blue spectra on the 113 region (where the gap is expected to decay over a shorter distance).

To understand the origin of the ~50 K *T*_C_, we undertook a detailed analysis of the crystal structure of film S3 in different regions of the film, in particular at interfacial regions. All the films are of very high epitaxial quality with a major fraction of *c*-214 and *a*-113 and some minor *a* axis–oriented 214 (*a*-214) (Fig. 3A).

The lattice parameters of the 214 films grown on STO were all in the range of *a* = 3.79 to 3.81 Å, *c* = 13.08 to 13.14 Å. *a* is slightly larger and *c* is slightly smaller than the bulk value of 214 (*a* = 3.794 Å), possibly because of a partial strain effect from the STO substrate. The minor *a*-214 is also observed in the planar transmission electron microscopy (TEM) image and exhibits an orthogonal pattern as shown in Fig. 3B.

Note S2 shows AFM topographic images of the same film. At first glance, the AFM images are similar to the planar TEM images as shown in Fig. 3B. However, at closer inspection, a different structure is seen, i.e., a much denser pattern of orthogonal grains is observed. Hence, these grains are not the minor *a*-214 grains observed in Fig. 3B. There is a 1:1 correspondence between the topography and current images in note S2, indicating that the film microstructure is made up of a major fraction of orthogonal conducting grains embedded in a nonconducting matrix.

Considering the XRD (x-ray diffraction), TEM, and AFM data altogether, the conducting grains correspond to *c*-214 and the nonconducting matrix is *a*-113. The structure differs from the idealized VAN nanopillar structure of Fig. 1B, which shows isolated nanopillars. Here, we have a highly faceted plate-like form of inclusions instead of nanopillars. Such a faceted structure should enable highly effective vertical strain control of *c*-214 by vertical epitaxy with *a*-113.

Further cross-sectional scanning TEM (STEM) analysis (Fig. 3C) reveals that the film is composed of *c*-214 and *a*-113, and the minor *a*-214 is present at some boundaries between *c*-214 and *a*-113 in the upper parts of the film. Roughly equivalent amounts of *c*-124 and *a*-113 grains are observed, consistent with the ratios of phases mixed in the target material, i.e., 214:113 = 2:3. In addition, the phases observed are the same as in the XRD plot of Fig. 3A and the planar TEM of Fig. 3B. Figure 3D shows a schematic structure of the film. The white-boxed areas (a and b) highlight regions that are probed more closely by cross-sectional STEM and spatial lattice parameter analyses to determine the origins of the 50 K phase.

We first examine Box a (Fig. 3E), which shows a nearly vertical *c*-214/*a*-113 boundary. In the region close to the substrate, *c* in the *a*-113 film is perfectly strained to the STO substrate and adopts the in-plane lattice parameter of STO, i.e., 3.905 Å. Hence, according to volume conservation, *a* in the *a*-113 film will be 57.993/(3.9052)^2^ = 3.803 Å. This value is slightly lower than the global *a* measured for *a*-113, which is 3.814 Å from XRD. A value of 3.814 Å is closer to the bulk value of 3.8189 Å for 113, consistent with the fact that there is some relaxation of *a* with film thickness.

The *c*-214/*a*-113 interface in Fig. 3E is stepped and not sharp and likely forms in this way to minimize interfacial energy in this high-energy interface from the dissimilar crystal structures. The *c*-214 grains are tilted by approximately 1° to 2° with respect to the horizontal plane. 113 grows *a* axis oriented on STO because *c* of 113 (*a* = 3.8189 Å and *c* = 3.97268 Å, for the fully oxidized tetragonal phase) more closely matches *a* of STO (3.905 Å). The phase grows coherently with the STO, as expected for these same crystal structures. *c*-214 grows on a very thin layer (two to three unit cells of 113 as shown in the left panel of Fig. 3E). *c*-214 grows more easily on *a*-113 than STO because 214 and 113 contain CuO_6_ building blocks, but it grows incoherently due to lattice misfit of the crystal structures.

In several regions of the vertical interface in Fig. 3E, domain matching epitaxy (DME) of exactly two unit cells of *c*-214 with seven unit cells of *a*-113 is observed. A schematic of the crystal structure matching is shown in Fig. 3F, with the interfacial region indicated by horizontal arrows. By knowing the value of *a* in 113, *c* in *c*-214 at the interface can be estimated using DME. Taking the lower *a* value of 3.803 Å, *c* in *c*-214 is calculated to be ~7 × 3.803/2 = 13.310 Å, which is much higher than the bulk value of 13.165 Å. *c* would be even higher toward the top of the film, where *a* in *a*-113 is larger owing to *a*-113 lattice relaxation with thickness.

**Fig. 4:**
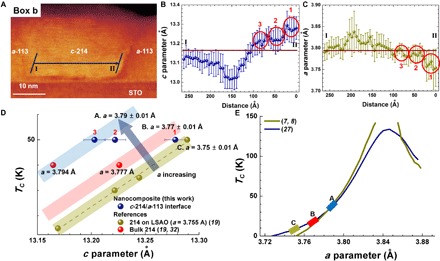
Structural analyses of 214:113. (**A**) STEM image of the *c*-214/*a*-113 boundary within white dashed Box b in Fig. 3 (C and D). The blue arrow from points I to II shows the horizontal distance where the lattice parameters were analyzed. The out-of-plane lattice parameters of the film were analyzed along the slanted lines that run parallel to the boundary and then averaged. (**B** and **C**) Lateral variation of the lattice parameters across the region from points I to II. The bars on each point reflect the variation in lattice parameters over the slanted regions in (A). The brown solid lines indicate the bulk lattice parameters of 214. (**D**) Relation of *T*_C_ and *c*. The averaged *c* values from regions 1, 2, and 3 in (B) and (C) are included in (D), as well as the data for bulk 214 (oxygenated and Sr-doped) and 214 films grown on LSAO substrates from (*19*, *32*). The projected influence of *a* on the plot [as determined from (E)] is shown schematically by the blue arrow. The yellow dashed line is a guide for 214 on LSAO (*19*). Yellow, red, and blue zones have the same slope as the guide for 214 on LSAO but with different *a*. (**E**) Maximum *T*_C_ versus *a* (*7*, *8*, *27*) for optimized cuprates. A strong dependence of *T*_C_ on *a* (and hence the in-plane Cu-O distance) is observed with a peak in *T*_C_ at *a* ~ 3.84 Å (*7*, *8*). Zones A, B, and C from (D) are included on the plot to show the good fit of the experimental data of this work to the “model” plot.

We now measure the local lattice parameters in a *c*-214 grain bounded by *a*-113 and compare these values with the above calculated value. Figure 4A shows a magnified STEM image of Box b of Fig. 3 (C and D). A horizontal line is shown from I to II in Fig. 4A, along which the lattice parameter calculations were made using an open source package, Atomap. Lines I to II represent a region in the *c*-214 grain that is not overlapped with the *a*-113 interface. Considering the nonvertical nature of the interfaces and the possible overlapping near the interfaces, points close to the *a*-113 interfaces on the left- and right-hand side could be influenced by the *a*-113 lattice. Point I was ~5 nm from the left-hand *a*-113 interface, whereas point II was ~2 nm from the right-hand *a*-113 interface. Analyses were undertaken to determine the lattice parameters spatially, as described in detail in Materials and Methods. This enabled the lattice parameters of each unit cell to be measured within ±0.01 Å.

At the region closest to the interface, i.e., in circled regions 1 to 3 in Fig. 4B, *c* in *c*-214 is very high, without *a* being reduced substantially in comparison with the bulk value of 3.794 Å, and this explains the ~50 K superconductivity (*19*). The enhancement of *c* by different amounts over a region of ~10 nm agrees with the broadened *R* (T) and the STM measurements (Fig. 2) and with the ~5% volume fraction of superconducting phase obtained from the magnetization measurements. The average *c* values in the three different regions of Fig. 4B are determined to be 13.28 ± 0.01 Å (region 1), 13.22 ± 0.01 Å (region 2), and 13.20 ± 0.01 Å (region 3), compared to the bulk value of *c* = 13.165 Å. The corresponding *a* values from the three different regions of Fig. 4C are 3.76 ± 0.01 Å, 3.78 ± 0.01 Å, and 3.79 ± 0.01 Å, compared to the bulk value of *a* = 3.794 Å.

The strong interfacial stretching of *c* to 13.28 ± 0.01 Å in region 1 is close to the calculated value of 13.310 Å from DME. The slightly smaller value is explained by the value not being measured right at the interface. Moving from the interface into the bulk of the *c*-214 grain, i.e., from II to I in Fig. 4A, *c* decays to a value of 13.02 ± 0.01 Å, i.e., lower than the bulk value (*c* = 13.1646 Å), but it rises again to the bulk value further into the *c*-214 grain.

*a* shows an inverse relation to *c*, as expected from elastic effects. The lattice parameters in *c*-214 have a spatial variation from the *a*-113 interface into the bulk of the *c*-214 grain. From very high *c* at the interface, there is dipping of *c* (and corresponding peaking of *a*) when moving into the *c*-214 grains. This can be explained by a residual stress state, compensating the strong vertical stretch near the interface. The higher-than-bulk *c* values will produce regions of higher *T*_C_ values, and the lower-than-bulk *c* values will produce regions of reduced *T*_C_ values.

Figure 4D shows *T*_C_ versus *c*. The *c* values were experimentally determined from Fig. 4B. Literature data for bulk 214 and films grown on LSAO (*19*, *32*) are also included. The *a* values determined from Fig. 4C are also included in Fig. 4D. The influence of *T*_C_ on the out-of-plane distance for cuprates was shown earlier in Fig. 1A, and the analogous *T*_C_ versus *a* plot is shown in Fig. 4E, together with the *a* values measured in Fig. 4C.

In Fig. 4D, for a given *c*, as one moves diagonally to the left of the diagram to the direction of the blue arrow, *T*_C_ increases rapidly with *a*. The yellow, red, and blue zones in Fig. 4D have different *a* values of 3.75 ± 0.01 Å, 3.77 ± 0.01 Å, and 3.79 ± 0.01 Å, respectively. The slope of the lines has been set at the experimentally determined slope for 214 on LSAO (*19*). The wider gap between the red and blue zones compared to the yellow and red zones in Fig. 4D is due to the increasing steepness of the *T*_C_ versus *a* plot (Fig. 4E) with increasing *a*.

Studies, so far, have considered the importance of either apical oxygen (and hence *c*) on *T*_C_ (Fig. 1A) or *a* on *T*_C_. Figure 4D reveals for the first time the great sensitivity of *T*_C_ to both *c* and *a*, and also indicates that a further enhancement of *T*_C_ would be possible in 214 if *c* and *a* were both further stretched. Hence, if *a* was optimized to be closer to the optimum 3.84 Å value (Fig. 4E), then this should push *T*_C_ even higher than the values we have observed in this work. Coherent growth of 214 on a structurally matched substrate with lattice parameters close to 3.84 Å is one possible approach to optimize *a*, as long as the VAN approach is also used to enhance *c* at the same time.

Last, this proof-of-concept work showing the importance of expansion of both *c* and *a* could be developed to give even larger volume fractions of 50 K superconductor than the ~5% obtained here. For example, by using faster growth rates, finer *c*-214 inclusions could be made (20 nm or below is desired so that *c*-214 is within the 10-nm strained interfacial region with the *c*-113 matrix), rather than the ~50-nm inclusions shown here. Achieving a finer microstructural size while maintaining high-quality epitaxy has been demonstrated previously in other VAN systems, even down to sizes as small as 2 to 5 nm (*33*).

## CONCLUSION

With the aim of vertically strain engineering *c* (and hence the apical distance) in *c* axis–oriented La_2_CuO_4+δ_ (*c*-214), we grew self-assembled, vertically aligned nanocomposite films of La_2_CuO_4+δ_ + LaCuO_3_ (113) on (001) STO. In a simple one-step process without any post-growth oxygenation using ozone and/or high-pressure oxygen as is normally used, 50 K superconducting material was produced in situ. The VAN approach does not reduce the in-plane lattice, as is normally the case for growth of 214 on standard LSAO substrates. The higher *T*_C_ phase is consistent with the presence of ~10-nm-wide regions of expansion of the out-of-plane 214 lattice (*c* parameter) produced at 214/113 vertical interfaces, spaced ~50 nm apart, of which there are billions per film. It is also consistent with the additional effect of relatively high in-plane lattice (*a* parameter). *T*_C_ equals the highest *T*_C_ of 50 K previously observed at one– to two–unit cell interfaces in ozone-annealed metal/superconductor bilayer heterostructures. Our proof-of-concept work represents an entirely new approach to 3D strain engineering of cuprates, giving the possibility to further enhance *T*_C_ values in 214 and other cuprate superconductors. This could be achieved by using a different strain controlling matrix material and/or different growth substrates, as well as larger volume fractions of higher *T*_C_ superconductor, giving finer (~20 nm or below) 214 inclusions, with minimal lateral strain relaxation.

## MATERIALS AND METHODS

### Target fabrication

We used a composite ceramic target of 214:113 with an atomic ratio (*n*:*m*) of 2:3. This ratio was chosen because we aimed to achieve relatively fine nanocolumns of *c*-214 so that, as much as possible, the column would be strained vertically by 113 without the column relaxing laterally to the bulk value. A smaller fraction of 214, and hence a lower *n*/*m* value, would give finer columns (*47*), but a very small value means that the columns would become discontinuous.

For reference, we made single-material targets of La_2_CuO_4_ (214) and LaCuO_3_ (113). Ceramic targets were synthesized by conventional solid-state reaction. A stoichiometric mixture of La_2_O_3_ and CuO was ground and subsequently calcined at 950°C for 12 hours under oxygen flow. After cooling down the mixture to room temperature, it was ground again, and this process was repeated three times. After this, the mixture was ground and pressed into a 1-inch-diameter pellet disc. The pellet was sintered at 1050°C for 12 hours under oxygen flow.

### Thin-film fabrication

More than 10 films (thickness, ~25 to 100 nm) were grown by PLD using a KrF excimer laser at a substrate temperature of 700°C with a repetition rate of 2 Hz and a fluence of 1.2 to 1.5 Jcm^−2^ with 0.13 to 0.14 mbar flowing O_2_. After growth, unless otherwise stated, the films were annealed in 500 mbar of O_2_ at 500°C for 60 min and then slowly cooled afterward (at a rate of –10°C/min) to room temperature while maintaining 500 mbar of O_2_ background pressure. The oxygenation conditions for the films are much less oxidizing than standard ozone annealing or high-pressure oxygen (>70 bar), which are normally required to make standard 214 films superconducting (*19*, *30*, *32*). This is expected because 113 is a highly oxidized phase with Cu in the Cu^3+^ state. Hence, it has the potential to oxidize 214 in the composite. TiO_2_-terminated (001)-oriented STO and 0.5 weight % Nb–doped STO (Nb:STO) (*a* = 3.905 Å) single-crystal substrates (CrysTec GmbH) were used.

### Characterization of structure and physical properties

Structural analysis of the films was done by XRD analysis using a high-resolution PANalytical Empyrean vertical diffractometer (Cu Kα radiation). Magnetic property measurements were performed using a superconducting quantum interference device (SQUID; Quantum Design) in a temperature range of 10 to 250 K.

Electrical transport measurements were performed on films grown on Nb:STO substrates using a four-probe configuration with two electrical contacts on Nb:STO and two contacts on a Pt electrode deposited on the thin-film surface with direct current magnetron sputtering. The inset of Fig. 2A shows a top-to-bottom measuring geometry.

XPS was performed using a SPECS PHOIBOS 150 electron energy analyzer with a monochromatic Al Kα x-ray source (*hv* = 1486.6 eV) and a total energy resolution of 500 meV. The BE was calibrated using a polycrystalline Au foil placed in electrical contact with the film surfaces after deposition, which simultaneously helped to avoid charging effects during XPS measurements.

### Scanning transmission electron microscopy

An FEI Titan G2 80-200 STEM with a Cs probe corrector, operated at 200 kV, was used in this study. The STEM images were recorded by using a high-angle annular dark-field detector.

### Lattice parameter determination

We used real-space image analysis to obtain maps of lattice parameters. The open source package Atomap (https://ascimaging.springeropen.com/articles/10.1186/s40679-017-0042-5, https://pypi.org/project/atomap/) was used to extract atomic column positions by fitting 2D Gaussian functions. Pairwise distances between neighboring columns were used to extract lattice parameters. Calibration was done using the distance between Sr columns of the STO substrate in the same field of view. The lattice parameter of STO was assumed to be 3.905 Å. We found that there was a systematic variation of the out-of-plane lattice parameter in the slow scan (out-of-plane) direction, which was likely caused by drift during image capture. To minimize this effect, profiles were taken in the horizontal (fast scan) direction without any vertical component. To cancel the systematic error in the out-of-plane lattice parameter, we calibrated the measured *c* using the values obtained from XRD. The averaged *c* value of *c*-214 from STEM is 13.15 Å, whereas the value from XRD is 13.14 Å for S3. We then calibrated *c* measured from STEM by using the difference between these two values. The systematic errors in the in-plane measurements were very small because they were measured in the fast scan direction. It is also important to consider the random errors in both the out-of-plane and in-plane directions. The random error was estimated by determining the range of lattice parameters measured in the STO substrate along the in-plane direction and was determined to be ±0.02 Å. Each data point in Fig. 4 (B and C) was an average of six measured points along the slanted lines of ~10 nm length parallel to the *c*-214/*a*-113 interface, as shown in Fig. 4A, giving an overall random error of ±0.02 Å/√6 = ±0.01 Å.

### Scanning tunneling microscopy

STM and STS measurements were carried out at 4.2 K using a cryogenic scanning tunneling microscope with a Pt-Ir tip operating in a clean He exchange gas environment. The differential conductance d*I*/d*V* versus the applied voltage *V* spectra were acquired with set current and bias-voltage values of 0.1 nA and 10 mV, respectively. A bias voltage of 100 mV, which is larger than the superconducting gap measured for the films, was used to acquire topographic images. The d*I*/d*V* spectra were fitted with the Dynes model by assuming unconventional d-wave symmetry of the superconducting order parameter. To determine the spatial variation of superconductivity, a gold capping layer was necessary to achieve good measurement stability over large scan lengths, as shown in (*46*).

## Supplementary Material

http://advances.sciencemag.org/cgi/content/full/5/4/eaav5532/DC1

Download PDF

## SUPPLEMENTARY MATERIALS

Supplementary material for this article is available at http://advances.sciencemag.org/cgi/content/full/5/4/eaav5532/DC1 Note S1. XPS data showing that O_2_ annealing (rather than O_3_ annealing) is sufficient to oxygenate La_2_CuO_4+δ_ in the nanocomposite films. Note S2. Conductance atomic force microscopy at room temperature. Note S3. Dependence of resistance versus temperature on bias current (100 and 500 μA). Note S4. Correlating tunneling spectra with topography and data reproducibility. Note S5. Fitting spectra measured in the Andreev spectroscopy regime. Fig. S1. XPS spectra (VB, O 1s, La 4d, and Cu 2p_3/2_) for films S4 and S5. Fig. S2. Atomic force microscopy images at room temperature for film S3. Fig. S3. *R* (T) with bias currents of 100 and 500 μA (top plot) and corresponding ZFC *M* (T) (bottom plot) for film S2. Fig. S4. Three tunneling spectra measured on sample S3. Fig. S5. Tunneling spectrum in the Andreev spectroscopy regime and fitting for sample S3. Reference (*48*)

## References

[1] Gao L., Xue Y. Y., Chen F., Xiong Q., Meng R. L., Ramirez D., Chu C. W., Eggert J. H., Mao H. K., Superconductivity up to 164 K in HgBa_2_Ca_*m*−1_Cu*_m_*O_2*m*+2+δ_ (*m*=1, 2, and 3) under quasihydrostatic pressures. Phys. Rev. B 50, 4260–4263 (1994). 10.1103/physrevb.50.4260 9976724

[2] Beasley M. R., Search for new very high temperature superconductors from an applications perspective. IEEE Trans. Appl. Supercond. 23, 7000304 (2013).

[3] MacManus-Driscoll J. L., Wimbush S. C., Future directions for cuprate conductors. IEEE Trans. Appl. Supercond. 21, 2495–2500 (2011).

[4] Orenstein J., Millis A. J., Advances in the physics of high-temperature superconductivity. Science 288, 468–474 (2000). 10775099 10.1126/science.288.5465.468

[5] Eisaki H., Kaneko N., Feng D. L., Damascelli A., Mang P. K., Shen K. M., Shen Z.-X., Greven M., Effect of chemical inhomogeneity in bismuth-based copper oxide superconductors. Phys. Rev. B 69, 064512 (2004).

[6] Fujita K., Noda T., Kojima K. M., Eisaki H., Uchida S., Effect of disorder outside the CuO_2_ planes on *T*_c_ of copper oxide superconductors. Phys. Rev. Lett. 95, 097006 (2005). 16197242 10.1103/PhysRevLett.95.097006

[7] Rao C. N. R., Ganguli A. K., Structure–property relationship in superconducting cuprates. Chem. Soc. Rev. 24, 1–7 (1995).

[8] Gadermaier C., Kabanov V. V., Alexandrov A. S., Stojchevska L., Mertelj T., Manzoni C., Cerullo G., Zhigadlo N. D., Karpinski J., Cai Y. Q., Yao X., Toda Y., Oda M., Sugai S., Mihailovic D., Strain-induced enhancement of the electron energy relaxation in strongly correlated superconductors. Phys. Rev. X 4, 011056 (2014).

[9] Zhang F. C., Rice T. M., Effective Hamiltonian for the superconducting Cu oxides. Phys. Rev. B 37, 3759–3761 (1988). 10.1103/physrevb.37.3759 9944993

[10] Sakakibara H., Usui H., Kuroki K., Arita R., Aoki H., Two-orbital model explains the higher transition temperature of the single-layer Hg-cuprate superconductor compared to that of the La-cuprate superconductor. Phys. Rev. Lett. 105, 057003 (2010). 20867949 10.1103/PhysRevLett.105.057003

[11] Scalapino D. J., A common thread: The pairing interaction for unconventional superconductors. Rev. Mod. Phys. 84, 1383–1417 (2012).

[12] Attfield J. P., Kharlanov A. L., McAllister J. A., Cation effects in doped La_2_CuO_4_ superconductors. Nature 394, 157–159 (1998).

[13] Gao W. B., Liu Q. Q., Yang L. X., Yu Y., Li F. Y., Jin C. Q., Uchida S., Out-of-plane effect on the superconductivity of Sr_2−*x*_Ba*_x_*CuO_3+δ_ withT_c_ up to 98 K. Phys. Rev. B 80, 094523 (2009).

[14] Suyolcu Y. E., Wang Y., Baiutti F., Al-Temimy A., Gregori G., Cristiani G., Sigle W., Maier J., van Aken P. A., Logvenov G., Dopant size effects on novel functionalities: High-temperature interfacial superconductivity. Sci. Rep. 7, 453 (2017). 28352070 10.1038/s41598-017-00539-4 PMC5428683

[15] Locquet J.-P., Perret J., Fompeyrine J., Mächler E., Seo J. W., Van Tendeloo G., Doubling the critical temperature of La_1.9_Sr_0.1_CuO_4_ using epitaxial strain. Nature 394, 453–456 (1998).

[16] Chen X.-J., Struzhkin V. V., Hemley R. J., Mao H.-k., Kendziora C., High-pressure phase diagram of Bi_2_Sr_2_CaCu_2_O_8+δ_ single crystals. Phys. Rev. B 70, 214502 (2004).

[17] Nakamura F., Goko T., Hori J., Uno Y., Kikugawa N., Fujita T., Role of two-dimensional electronic state in superconductivity in La_2−*x*_Sr*_x_*CuO_4_. Phys. Rev. B 61, 107–110 (2000).

[18] Gozar A., Logvenov G., Fitting Kourkoutis L., Bollinger A. T., Giannuzzi L. A., Muller D. A., Bozovic I., High-temperature interface superconductivity between metallic and insulating copper oxides. Nature 455, 782–785 (2008). 18843365 10.1038/nature07293

[19] Butko V. Y., Logvenov G., Božović N., Radović Z., Božović I., Madelung strain in cuprate superconductors - A route to enhancement of the critical temperature. Adv. Mater. 21, 3644–3648 (2009).

[20] Pavarini E., Dasgupta I., Saha-Dasgupta T., Jepsen O., Andersen O. K., Band-structure trend in hole-doped cuprates and correlation with *T*_*c* max_. Phys. Rev. Lett. 87, 47003 (2001). 10.1103/PhysRevLett.87.047003 11461638

[21] Smadici S., Lee J. C. T., Wang S., Abbamonte P., Logvenov G., Gozar A., Deville Cavellin C., Bozovic I., Superconducting transition at 38 K in insulating-overdoped La_2_CuO_4_−La_1.64_Sr_0.36_CuO_4_ superlattices: Evidence for interface electronic redistribution from resonant soft X-ray scattering. Phys. Rev. Lett. 102, 107004 (2009). 19392148 10.1103/PhysRevLett.102.107004

[22] Wu J., Pelleg O., Logvenov G., Bollinger A. T., Sun Y.-J., Boebinger G. S., Vanević M., Radović Z., Božović I., Anomalous independence of interface superconductivity from carrier density. Nat. Mater. 12, 877–881 (2013). 23913171 10.1038/nmat3719

[23] Saito Y., Nojima T., Iwasa Y., Highly crystalline 2D superconductors. Nat. Rev. Mater. 2, 16094 (2016).

[24] Caviglia A. D., Gariglio S., Reyren N., Jaccard D., Schneider T., Gabay M., Thiel S., Hammerl G., Mannhart J., Triscone J.-M., Electric field control of the LaAlO_3_/SrTiO_3_ interface ground state. Nature 456, 624–627 (2008). 19052624 10.1038/nature07576

[25] Radović Z., Božović N., Božović I., Photoinduced expansion of cuprate superconductors: Evidence of strong electron-lattice coupling. Phys. Rev. B Condens. Matter Mater. Phys. 77, 092508 (2008).

[26] He S., He J., Zhang W., Zhao L., Liu D., Liu X., Mou D., Ou Y.-B., Wang Q.-Y., Li Z., Wang L., Peng Y., Liu Y., Chen C., Yu L., Liu G., Dong X., Zhang J., Chen C., Xu Z., Chen X., Ma X., Xue Q., Zhou X. J., Phase diagram and electronic indication of high-temperature superconductivity at 65 K in single-layer FeSe films. Nat. Mater. 12, 605–610 (2013). 23708329 10.1038/nmat3648

[27] Bianconi A., Agrestini S., Bianconi G., Di Castro D., Saini N. L., A quantum phase transition driven by the electron lattice interaction gives high *T*_C_ superconductivity. J. Alloys Compd. 317–318, 537–541 (2001).

[28] Naito M., Tsukada A., Greibe T., Sato H., Phase control in La-214 epitaxial thin films. Proc. SPIE 4811, 140–154 (2002).

[29] Tsukada A., Greibe T., Naito M., Phase control of La_2_CuO_4_ in thin film synthesis. Phys. Rev. B Condens. Matter Mater. Phys. 66, 1845151–1845155 (2002).

[30] Trofimov I. E., Johnson L. A., Ramanujachary K. V., Guha S., Harrison M. G., Greenblatt M., Cieplak M. Z., Lindenfeld P., Growth and properties of La_2−*x*_Sr*_x_*CuO_4_ films. Appl. Phys. Lett. 65, 2481–2483 (1994).

[31] He J., Klie R. F., Logvenov G., Bozovic I., Zhu Y., Microstructure and possible strain relaxation mechanisms of La_2_CuO_4+δ_ thin films grown on LaSrAlO_4_ and SrTiO_3_ substrates. J. Appl. Phys. 101, 073906 (2007).

[32] Meyer T. L., Jiang L., Park S., Egami T., Lee H. N., Strain-relaxation and critical thickness of epitaxial La_1.85_Sr_0.15_CuO_4_ films. APL Mater. 3, 126102 (2015).

[33] MacManus-Driscoll J. L., Suwardi A., Wang H., Composite epitaxial thin films: A new platform for tuning, probing, and exploiting mesoscale oxides. MRS Bull. 40, 933–942 (2015).

[34] Karppinen M., Yamauchia H., Suematsu H., Isawa K., Nagano M., Itti R., Fukunaga O., Control on the copper valence and properties by oxygen content adjustment in the LaCuO_3−*y*_ system (0≤*y*≤0.5). J. Solid State Chem. 222, 213–222 (1997).

[35] Czyżyk M. T., Sawatzky G. A., Local-density functional and on-site correlations: The electronic structure of La_2_CuO_4_ and LaCuO_3_. Phys. Rev. B 49, 14211–14228 (1994). 10.1103/physrevb.49.14211 10010501

[36] Bringley J. F., Scott B. A., La Placa S. J., Mcguire T. R., Mehran F., McElfresh M. W., Cox D. E., Structure and properties of the LaCuO_3−δ_ perovskites. Phys. Rev. B 47, 15269–15275 (1993). 10.1103/physrevb.47.15269 10005901

[37] Imada M., Fujimori A., Tokura Y., Metal-insulator transitions. Rev. Mod. Phys. 70, 1039–1263 (1998).

[38] Engelmann J., Grinenko V., Chekhonin P., Skrotzki W., Efremov D. V., Oswald S., Iida K., Hühne R., Hänisch J., Hoffmann M., Kurth F., Schultz L., Holzapfel B., Strain induced superconductivity in the parent compound BaFe_2_As_2_. Nat. Commun. 4, 2877 (2013). 24309386 10.1038/ncomms3877

[39] Moodenbaugh A. R., Xu Y., Suenaga M., Folkerts T. J., Shelton R. N., Superconducting properties of La_2−x_Ba_x_CuO_4_. Phys. Rev. B 38, 4596–4600 (1988). 10.1103/physrevb.38.4596 9946848

[40] Lv B., Deng L., Gooch M., Wei F., Sun Y., Meen J. K., Xue Y.-Y., Lorenz B., Chu C.-W., Unusual superconducting state at 49 K in electron-doped CaFe_2_As_2_ at ambient pressure. Proc. Natl. Acad. Sci. U.S.A. 108, 15705–15709 (2011). 21911404 10.1073/pnas.1112150108 PMC3179039

[41] Kato T., Okitsu S., Sakata H., Inhomogeneous electronic states of La_2–*x*_Sr*_x_*CuO_4_ probed by scanning tunneling spectroscopy. Phys. Rev. B Condens. Matter Mater. Phys. 72, 144518 (2005).

[42] Boyer M. C., Wise W. D., Chatterjee K., Yi M., Kondo T., Takeuchi T., Ikuta H., Hudson E. W., Imaging the two gaps of the high-temperature superconductor Bi_2_Sr_2_CuO_6+*x*_. Nat. Phys. 3, 802–806 (2007).

[43] Alpern H., Katzir E., Yochelis S., Katz N., Paltiel Y., Millo O., Unconventional superconductivity induced in Nb films by adsorbed chiral molecules. New J. Phys. 18, 113048 (2016).

[44] Dagan Y., Beck R., Greene R. L., Dirty superconductivity in the electron-doped cuprate Pr_2–*x*_Ce*_x_*CuO_4–δ_: Tunneling study. Phys. Rev. Lett. 99, 147004 (2007). 17930707 10.1103/PhysRevLett.99.147004

[45] Tanaka Y., Kashiwaya S., Theory of tunneling spectroscopy of *d*-wave superconductors. Phys. Rev. Lett. 74, 3451–3454 (1995). 10058204 10.1103/PhysRevLett.74.3451

[46] Levi Y., Millo O., Sharoni A., Tsabba Y., Leitus G., Reich S., Evidence for localized high-*T*_C_ superconducting regions on the surface of Na-doped WO_3_. Europhys. Lett. 51, 564–570 (2000).

[47] MacManus-Driscoll J., Suwardi A., Kursumovic A., Bi Z., Tsai C.-F., Wang H., Jia Q., Lee O. J., New strain states and radical property tuning of metal oxides using a nanocomposite thin film approach. APL Mater. 3, 062507 (2015).

[48] Dynes R. C., Narayanamurti V., Garno J. P., Direct measurement of quasiparticle-lifetime broadening in a strong-coupled superconductor. Phys. Rev. Lett. 41, 1509–1512 (1978).

