# Supplementary material for: 3D strain-induced superconductivity in La2CuO4+δ using a simple vertically aligned nanocomposite approach
Source: Sci Adv. 2019 Apr 26;5(4):eaav5532. doi: 10.1126/sciadv.aav5532 (PMC6486216; doi:10.1126/sciadv.aav5532)
Supplement: http://advances.sciencemag.org/cgi/content/full/5/4/eaav5532/DC1 [file supp_5_4_eaav5532__index.html]

Science Advances | Science Advances

## Supplementary Materials

**This PDF file includes:**

- Note S1. XPS data showing that O2 annealing (rather than O3 annealing) is sufficient to oxygenate La2CuO4+δ in the nanocomposite films.
- Note S2. Conductance atomic force microscopy at room temperature.
- Note S3. Dependence of resistance versus temperature on bias current (100 and 500 μA).
- Note S4. Correlating tunneling spectra with topography and data reproducibility.
- Note S5. Fitting spectra measured in the Andreev spectroscopy regime.
- Fig. S1. XPS spectra (VB, O 1s, La 4d, and Cu 2p3/2) for films S4 and S5.
- Fig. S2. Atomic force microscopy images at room temperature for film S3.
- Fig. S3. *R* (T) with bias currents of 100 and 500 μA (top plot) and corresponding ZFC *M* (T) (bottom plot) for film S2.
- Fig. S4. Three tunneling spectra measured on sample S3.
- Fig. S5. Tunneling spectrum in the Andreev spectroscopy regime and fitting for sample S3.
- Reference (*48*)

Download PDF

**Files in this Data Supplement:**

- Adobe PDF - aav5532\_SM.pdf
