# Supplementary material for: 3D strain-induced superconductivity in La2CuO4+δ using a simple vertically aligned nanocomposite approach
Source: Sci Adv. 2019 Apr 26;5(4):eaav5532. doi: 10.1126/sciadv.aav5532 (PMC6486216; doi:10.1126/sciadv.aav5532)
Supplement: Download PDF [file aav5532_SM.pdf]

Science Advances
AAAS

advances.sciencemag.org/cgi/content/full/5/4/eaav5532/DC1

Supplementary Materials for

3D strain-induced superconductivity in $La_2CuO_{4+\delta}$ using a simple vertically aligned nanocomposite approach

Eun-Mi Choi*, Angelo Di Bernardo, Bonan Zhu, Ping Lu, Hen Alpern, Kelvin H. L. Zhang, Tamar Shapira, John Feighan, Xing Sun, Jason Robinson, Yossi Paltiel, Oded Millo, Haiyan Wang, Quanxi Jia, Judith L. MacManus-Driscoll*

*Corresponding author. Email: emc63@cam.ac.uk (E.-M.C.); jld35@cam.ac.uk (J.L.M.-D.)

Published 26 April 2019, *Sci. Adv.* **5**, eaav5532 (2019)
DOI: 10.1126/sciadv.aav5532

This PDF file includes:

Note S1. XPS data showing that O_2 annealing (rather than O_3 annealing) is sufficient to oxygenate $La_2CuO_{4+\delta}$ in the nanocomposite films.
Note S2. Conductance atomic force microscopy at room temperature.
Note S3. Dependence of resistance versus temperature on bias current (100 and 500 μA).
Note S4. Correlating tunneling spectra with topography and data reproducibility.
Note S5. Fitting spectra measured in the Andreev spectroscopy regime.
Fig. S1. XPS spectra (VB, O 1s, La 4d, and Cu $2p_{3/2}$) for films S4 and S5.
Fig. S2. Atomic force microscopy images at room temperature for film S3.
Fig. S3. *R* (T) with bias currents of 100 and 500 μA (top plot) and corresponding ZFC *M* (T) (bottom plot) for film S2.
Fig. S4. Three tunneling spectra measured on sample S3.
Fig. S5. Tunneling spectrum in the Andreev spectroscopy regime and fitting for sample S3.
Reference (*48*)

Supplementary Materials

Note S1. XPS data showing that O_2 annealing (rather than O_3 annealing) is sufficient to oxygenate $La_2CuO_{4+\delta}$ in the nanocomposite films.

To show that simple O_2 annealing, rather than O_3 annealing as is normally the case for plain 214 films, is sufficient to provide hole doping for superconductivity, x-ray photoemission spectroscopy (XPS) was undertaken for two nanocomposite films, one cooled without post-annealing in O2 after growth (S5), and the other with post-annealing at a *p*O2 of 500 mbar for 1 hour after growth (S3).

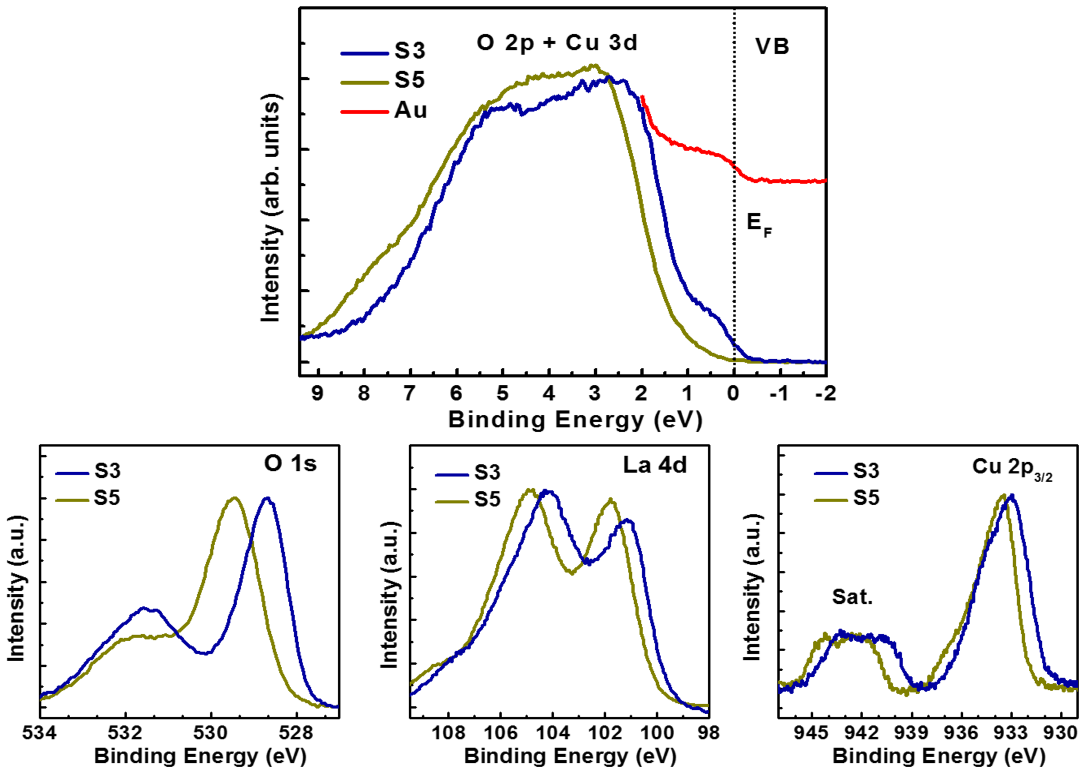

Fig. S1. XPS spectra (VB, O 1s, La 4d, and Cu $2p_{3/2}$) for films S4 and S5. XPS valence band spectra for films S4 and S5, along with the spectrum from a Au foil for energy calibration (red curve). There is no intensity at the Fermi level (Ef) for S5, whereas a clear density of state appears for S3, indicating an "insulator-to-metal" transition after annealing in O_2. The binding energies of O1s and La 4d for the S3 shifts by around 0.8 eV towards a lower binding energy with respect to those of S5. This BE shift is assigned to a downward shift of the Fermi level, which has been observed in many hole-doped transition metal oxides and hole-doped cuprate oxides (*37*). This indicates that the superconducting film is hole-doped.

Since we did not carry out any process for charge carrier doping, i.e. superoxygenation by ozone annealing or chemical doping, it is clear that the 113 phase in the nanocomposite plays an important role in enabling superoxygenation of the film. This is not surprising as Cu is in the highly oxidized Cu^{3+} state in 113.

Note S2. Conductance atomic force microscopy at room temperature.

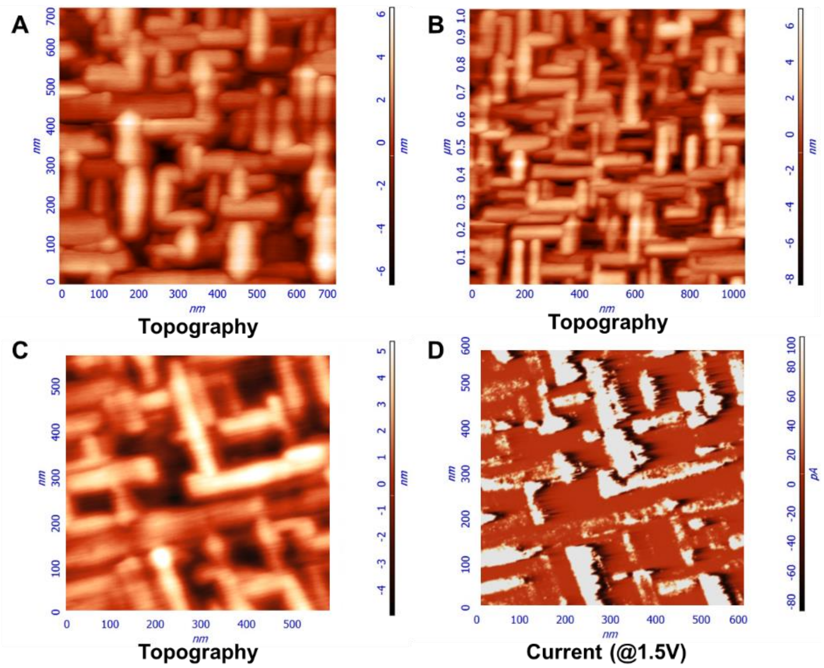

Fig. S2. Atomic force microscopy images at room temperature for film S3. (**A** and **B**) Typical AFM topographic images measured on sample S3 using the constant-pressure contact mode. (**C** and **D**) Conductance Atomic Force Microscopy (C-AFM) measurement on the same sample. (C) The topography and (D) the current map, were both measured at a bias of 1.5 V. Note that current is observable only at localized regions. The current map is correlated with the topography image, and indicates conducting orthogonal grains embedded in an insulating matrix. The conducting regions are consistent with the *c*-214 grains in which superconductivity develops at lower temperatures.

Note S3. Dependence of resistance versus temperature on bias current (100 and 500 μA).

The dependence of resistance versus temperature on bias current I for film S2 (top plot) in a top to bottom measurement which matches the magnetic transition (bottom plot). On-going from low currents (red curve) to higher currents (blue curve) a reduced T_C of 40 K is observed. We note that the broad transition in these films can be due to several reasons including an inhomogeneous variation of strain at the *a*-113/*c*-214 interfaces resulting in regions with different T_C onset or a non-continuous superconducting path leading to percolation effects as explained in the main text. The presence of an insulating 113-layer between the superconducting regions and the substrate (see STEM image in Fig. 3E of the main text) with varying thickness across the sample can also account for a broad superconducting transition. In particular, electron tunneling through this insulating barrier, which is placed along the current path in the top-to-bottom measurement configuration, becomes more efficient as the bias current is increased. This can also explain the higher temperature offset of the superconducting transition observed in fig. S3 when the bias current is increased from 100 μA (red curve) to 500 μA (blue curve).

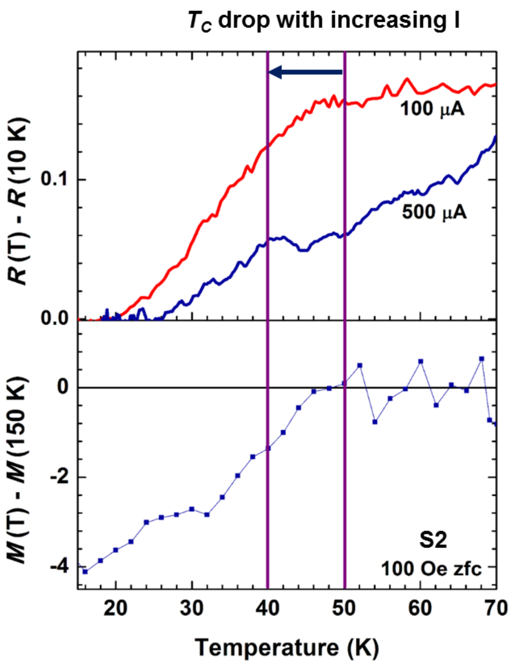

Fig. S3. *R* (T) with bias currents of 100 and 500 μA (top plot) and corresponding ZFC *M* (T) (bottom plot) for film S2. The estimated T_C value from these transport measurements is 50 K.

Note S4. Correlating tunneling spectra with topography and data reproducibility.

Figure S4 presents the only case in which we were able to show some correlation between topography and spectroscopy on an uncoated sample. The two spectra shown in fig. S4A (black and blue curves) were measured approximately in the middle of the nanostructure observed in the topographic image presented in the inset. These two spectra were measured after two consecutive tip-to-sample approaches, manifesting a good degree of data reproducibility. The spectrum shown in fig. S4B (black curve) was measured in another location, closer to what appears to be a boundary showing one of the widest gaps observed. The simulated spectra (dashed red curves) were calculated using the Tanaka and Kashiwaya model for tunneling to a d-wave superconductor by (*45*). The fitting parameters in fig. S4A are: $\Delta = 4.9$ meV (gap), $Z = 6$ (the dimensionless tunnel barrier strength – confirming here the bona-fide tunneling condition) ; $\Gamma = 0.47$ meV (the Dynes life-time broaden parameter (*48*)), and in fig. S4B: $\Delta = 8$ meV, $Z = 10$, $\Gamma = 3.2$ meV.

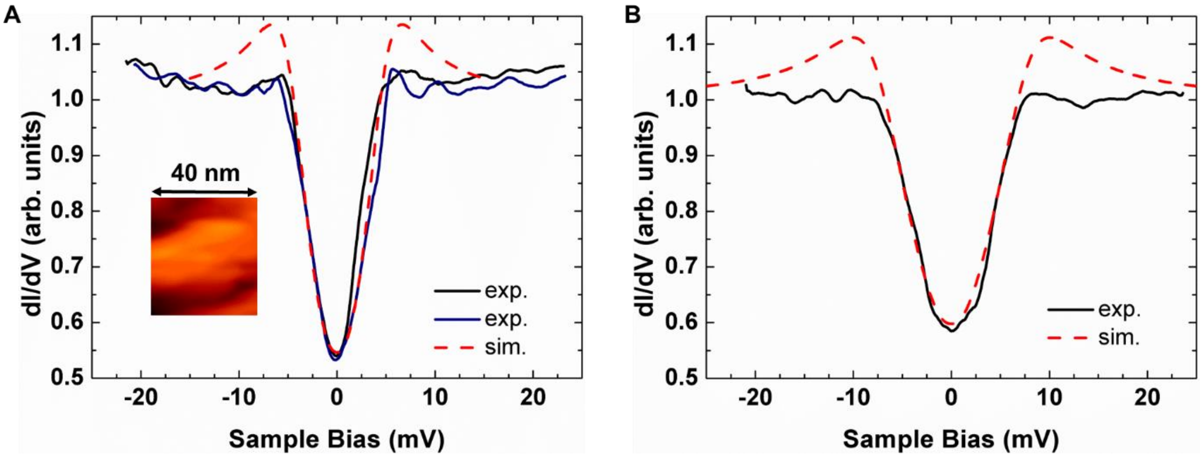

Fig. S4. Three tunneling spectra measured on sample S3. The two in (**A**) (blue and black curves) were measured approximately in the middle of the area shown by the STM topographic map (inset), exhibiting a nanostructured inclusion. (**B**) Another spectrum (black curve) measured in another location, closer to the upper boundary. The dashed red curves are fits (simulated spectra) calculated as detailed in the text, with gaps of 5 meV (A) and 8 meV (B).

Note S5. Fitting spectra measured in the Andreev spectroscopy regime.

In some cases we were able to form stable high-transparent junctions between the tip and the sample. The measured spectra then could be fitted to the Tanaka and Kashiwaya model for tunneling into a *d*-wave superconductor, but with low Z values, corresponding to the Andreev spectroscopy regime. One such spectrum is shown in fig. S5.

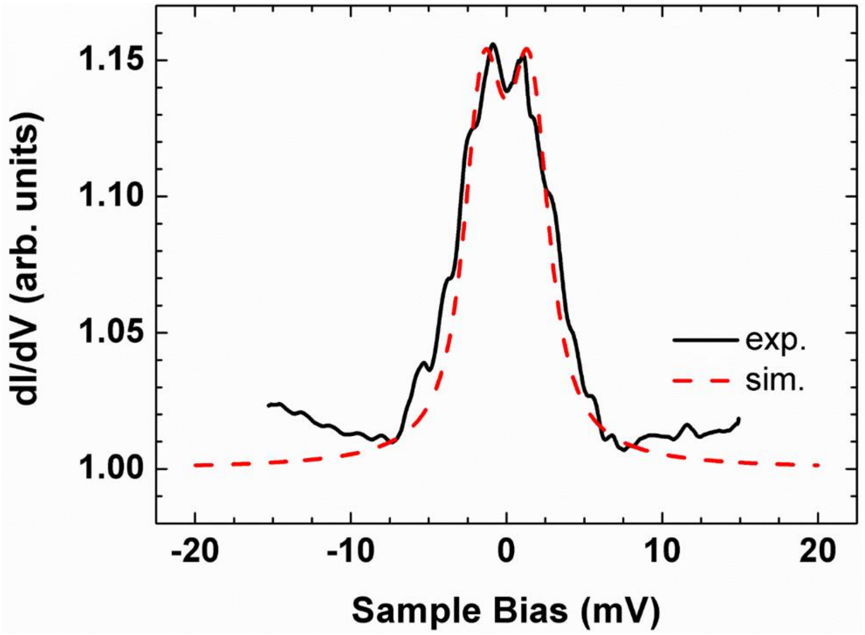

Fig. S5. Tunneling spectrum in the Andreev spectroscopy regime and fitting for sample S3. The spectrum was measured in the Andreev spectroscopy regime (low Z) regime (black curve). The fitting was to a simulated d-wave spectrum, (dashed red curve). The fitting parameters are: $\Delta = 2.5$ meV ; $Z = 0.6$; $\Gamma = 0.68$ meV.
